# The Battle against Cardiogenic Shock

**DOI:** 10.3390/jcm11236958

**Published:** 2022-11-25

**Authors:** Eldad Rahamim, Shemy Carasso, Offer Amir, Gabby Elbaz-Greener

**Affiliations:** 1 Department of Cardiology, Hadassah Medical Center, The Faculty of Medicine, Hebrew University of Jerusalem, Jerusalem 91120, Israel; 2 Shaare Zedek Medical Center, Hebrew University of Jerusalem, Jerusalem 9103102, Israel; 3 The Azrieli Faculty of Medicine in the Galilee, Bar-Ilan University, Safed 1311502, Israel

Cardiogenic shock (CS) is a life-threatening condition characterized by hypoperfusion and hypoxia caused by low cardiac output. The ESC guidelines focus on the clinical syndrome of CS, which includes systolic blood pressure (SBP) < 90 mmHg, clinical signs of hypoperfusion, which include cold extremities, oliguria, altered mental status, dizziness and laboratory signs, such as metabolic acidosis, elevated serum lactate and elevated serum creatinine levels. Additional definitions of CS in clinical trials include a cardiac index under 2.2 L·min^−1^·m^−2^, left ventricular ejection fraction of ≤40%, or pulmonary capillary wedge pressure (PCWP) ≥ 15 mmHg [1].

The most common hemodynamic status is low cardiac output, but PCWP, volume, and systemic vascular resistance (SVR) may vary, as CS can coexist with other types of shocks, such as septic, hemorrhagic and obstructive shocks [2]. Patients can be classified into the following five stages: stage A (“At risk”, without symptoms or signs of shock), stage B (“Beginning”, with relative hypotension or tachycardia without evidence of hypoperfusion), stage C (“Classic” CS), stage D (“Doom”, deteriorating CS), and stage E, (“Extremis”, in which patients experience cardiac arrest and require mechanical circulatory support (MCS) or cardiopulmonary resuscitation (CPR)) [1]. At this stage of shock, patients do not respond to fluid resuscitation and usually require pharmacological intervention and mechanical support. The vascular compensatory mechanism, including vasoconstriction, might worsen the shock by increasing the afterload on the failing heart. In addition, the inflammatory process that accompanies myocardial infarctions often leads to high cytokine levels with detrimental outcomes [3]. 

Acute myocardial infarction (MI) is the leading cause of this fatal condition. Mortality rates were as high as 80% before early revascularization was the standard of care. Mortality rates decreased to 50% in the contemporary revascularization era [4]. Today, patients with CS should be transferred as soon as possible to a tertiary center with full percutaneous coronary intervention capabilities. No significant evidence is available regarding fibrinolysis in CS. 

About 80% of CS cases are caused by acute coronary syndrome (ACS), which is associated with hemodynamic instability; however, CS may also be caused by valvular, pericardial, or electrical pathologies, complicating ACS, or occur without relation to ACS [5]. Between 2003 and 2010, there was an increase in the incidence of CS in STEMI patients from 6.5% to 10.1%. During the same period, the mortality rates decreased from 44.6% to 33.8%, although the rates decreased less in patients over 75 years old [6]. 

Evaluation of CS patients includes a thorough history and physical examination, looking for triggers that might explain the new onset/decompensation. Lab work might include the use of biomarkers to assess the level of myonecrosis, such as troponin. Natriuretic peptides, such as NT-Pro-BNP, are also helpful and are associated with mortality [7]. Chest X-rays might reveal pulmonary congestion or an enlarged heart and may help to rule out alternative diagnoses. Resting ECG is crucial in diagnosing ACS, especially the ST segment elevation type, but it can provide important insights into other conditions. Transthoracic echocardiography is essential in the diagnosis of CS, as it allows us to assess ventricular function (both global and regional), forward stroke volume, valvular dysfunction and pericardial fluid and function. Point-of-care US (POCUS) plays a major role in the acute management of these patients. This tool allows us to assess cardiac output, fluid status via vena cava measurement and respiratory collapse and pulmonary congestion through lung ultrasound. The advantage of POCUS is the availability and ease of use, allowing for serial evaluation and monitoring.

Mortality is high in patients with CS. In the SHOCK trial, it was reported that six years after CS hospitalization, 38% of the patients died [8]. Patients with CS had higher mortality rates in the first 60 days after discharge than non-CS acute MI patients. Patients with CS are faced with significant morbidity rates and high rehospitalization rates (up to 60%) [9]. A third to half of CS patients experience significant morbidity rates post-discharge [10]. Scarce data are available regarding the prognosis of CS patients of non-ACS causes.

An increase in early revascularization and in the use of intra-aortic balloon pumps (IABP) was noted between 2003 and 2010. The use of early invasive approaches might be the reason for the decrease in mortality rates [6]. The GUSTO-1 trial showed that the use of tissue plasminogen activator and streptokinase reduced the rates of CS, but did not have a significant effect on mortality [11]. Early invasive therapy is still the best option for treating CS patients, as observed in the SMASH study [12]. In the SHOCK trial, decreased mortality rates were reported for the early revascularization group at 6 and 12 months. Moreover, patients with successful percutaneous coronary intervention (PCI) with stents had lower mortality rates compared to those who did not [8]. Early revascularization is recommended in patients with multivessel disease and CS [13]

In the CULPRIT-SHOCK trial, the composite risk of death or renal replacement therapy was lower among CS patients that were invasively treated only in the culprit lesion as opposed to all lesions [14]. In the SHOCK trial, similar 1-year mortality rates between patients treated by PCI or by coronary artery bypass graft surgery (CABG) were reported. Today, most CS patients with MI are treated with PCI, not CABG.

In some instances, pharmacological therapy is insufficient, and CS patients need mechanical support. Mechanical support devices can be inserted percutaneously or surgically. These are removed once the heart recuperates. The INTERMACS profiles for CS classify patients based on their severity, with INTERMACS 1 representing “Crash and Burn” patients that are the sickest. Patients with INTERMACS 1 and 2 have the lowest survival rates and there has been a decline in the use of mechanical support in these patients.

The IABP is the most widely used mechanical device. The IABP-SHOCK II trial found no difference in the 30-day mortality rates or in the other secondary outcomes of patients [10,15]. This meant that IABP became a class IIIA recommendation for patients with CS in the ESC guidelines.

Percutaneous mechanical circulatory support (MCS) includes a few devices that are on the market. The data on these devices are still limited. These devices have been compared to IABP and there have been mixed results in these trials. In the US registry USpella, it was reported that patients treated with impella prior to coronary intervention had higher survival rates and hospital discharge rates [16]. Other trials showed no mortality benefit compared to IABP. In the IMPRESS-in-Severe-SHOCK trial, 48 patients with CS were randomized to a group with impella or IABP. This trial was underpowered. There was no difference in all-cause mortality at 30 days [17].

Extracorporeal membrane oxygenation (ECMO) can support the respiratory and cardiovascular systems. In patients with CS, veno-arterial ECMO is used. ECMO is mainly beneficial for patients with CS as a result of reversible causes [18]. A recent meta-analysis showed the benefit of ECMO in SC patients [19]. Patients with refractory cardiogenic shock resulting from acute valvular disease can benefit from early percutaneous intervention. Transcatheter edge-to-edge repair of mitral regurgitation was also found to improve survival of patients [20].

The mortality rates of patients with CS have not changed dramatically in the past few decades. Nevertheless, a new approach to the management of revascularization in patients with CS is included in the guideline’s recent updates. Clinical studies that focus on the management of CS are complicated, leading to scarce evidence. Large-scale registries are urgently required in order to improve the management of this fatal condition. With that in mind, we conclude that the recent evidence shows that there are more invasive tools to discover in the battle against CS.
